# Mapping brain functional networks topological characteristics in new daily persistent headache: a magnetoencephalography study

**DOI:** 10.1186/s10194-023-01695-x

**Published:** 2023-12-06

**Authors:** Dong Qiu, Zhaoli Ge, Yanliang Mei, Wei Wang, Zhonghua Xiong, Xiaoshuang Li, Ziyu Yuan, Peng Zhang, Mantian Zhang, Xin Liu, Yaqing Zhang, Xueying Yu, Hefei Tang, Yonggang Wang

**Affiliations:** 1 Department of Neurology, Headache Center, Beijing Tiantan Hospital, Capital Medical University, No.119 South Fourth Ring West Road, Fengtai District, Beijing, 100070 China; 2 Department of Neurology, Shenzhen Second People’s Hospital, Shenzhen, 518000 Guangdong China

**Keywords:** New daily persistent headache, Magnetoencephalography, Functional connectivity, Network topology

## Abstract

**Background:**

The brain functional network topology in new daily persistent headache (NDPH) is not well understood. In this study, we aim to assess the cortical functional network topological characteristics of NDPH using non-invasive neural signal recordings.

**Methods:**

Resting-state magnetoencephalography (MEG) was used to measure power fluctuations in neuronal oscillations from distributed cortical parcels in 35 patients with NDPH and 40 healthy controls (HCs). Their structural data were collected by 3T MRI. Functional connectivity (FC) of neural networks from 1 to 80 Hz frequency ranges was analyzed with topographic patterns and calculated network topological parameters with graph theory.

**Results:**

In the delta (1–4 Hz) and beta (13–30 Hz) bands, the lateral occipital cortex and superior frontal gyrus FC were increased in NDPH groups compared to HCs. Graph theory analysis revealed that the NDPH had significantly increased global efficiency in the delta band and decreased nodal clustering coefficient (left medial orbitofrontal cortex) in the theta (4–8 Hz) band. The clinical characteristics had a significant correlation with network topological parameters. Age at onset of patients showed a positive correlation with global efficiency in the delta band. The degree of depression of patients showed a negative correlation with the nodal clustering coefficient (left medial orbitofrontal cortex) in the theta band.

**Conclusion:**

The FC and topology of NDPH in brain networks may be altered, potentially leading to cortical hyperexcitability. Moreover, medial orbitofrontal cortex is involved in the pathophysiological mechanism of depression in patients with NDPH. Increased FC observed in the lateral occipital cortex and superior frontal gyrus during resting-state MEG could serve as one of the imaging characteristics associated with NDPH.

## Introduction

New daily persistent headache (NDPH) was initially introduced by Vanast (1986) as a benign form of headache, characterized by persistent headache with a particular temporal profile as it starts one day with a clearly remembered onset and continues in a daily pattern without remitting [1, 2]. Although epidemiological studies on the prevalence of NDPH are not abundant, the disorder is a rare condition, with the only study meeting inclusion criteria finding a prevalence of 0.03% [3]. Persistent headache seriously affects the quality of life of patients. However, the pathogenesis of NDPH is still unclear [4].

Resting-state functional networks reflect the functional organization of the brain [5]. Magnetic resonance imaging (MRI) resting-state activity exposes significant aberrant functional connectivity (FC) in multiple brain regions among patients with NDPH [6]. Even though several alterations in brain functional connections have been pinpointed in NDPH, there is still a need for a comprehensive examination of the entire brain's connectivity network and its topological characteristics [7]. Recent developments in neuroscience emphasize that brain architecture is a combination of tightly connected networks that control diverse brain functions [8]. A deeper understanding of how network organization can be altered or disrupted during NDPH is important to clarify the pathophysiology of NDPH.

Graph theory-based complex brain network analysis provides a powerful framework to examine the topological architecture of brain networks. In graph theory, the brain is a digital network of nodes and edges. In the network, nodes represent brain regions, and edges represent FC between brain regions [9]. In neuroscience, graph theory has been widely used to understand how the segregation and integration of brain regions participate in the pathophysiological processes of diseases [10].

Magnetoencephalography (MEG), which has been used in the study of neural networks in headache, is an advanced technique that can noninvasively collect neuronal magnetic activity [11]. Compared with electroencephalography (EEG), MEG has a higher spatial resolution. Moreover, neuromagnetic signals can more directly observe neuronal activity than bold signals of functional MRI. Graph theory analysis of MEG has been widely used in neuroscience to observe the intrinsic characteristics of functional networks. Characteristics in resting state can be used as markers reflecting the progression of diseases, such as Alzheimer’s disease and Parkinson’s disease [12, 13]. Therefore, we applied resting-state MEG to study whether there were abnormalities in the neural network of patients with NDPH. In addition, we evaluated whether there was a correlation between the clinical characteristics of patients with NDPH and the network topological parameters. Our study provides clues and evidence for exploring the pathophysiological mechanism of NDPH.

## Methods

### Study population

From May 2020 to August 2023, 40 patients with NDPH and 43 healthy controls (HCs) were recruited from the Headache Department, Neurology Centre, Beijing Tiantan Hospital, Capital Medical University. Each recruited patient needed to be diagnosed with NDPH by two specialist neurologists. The inclusion criteria of NDPH group: (1) Satisfied the diagnostic criteria of NDPH according to ICHD-3 criteria; (2) Ages 18 to 70 years; (3) None of the patients enrolled had been prophylactically treated for NDPH for at least 3 months. The exclusion criteria of NDPH group: (1) Combined with other types of primary headache or major systemic diseases; (2) Inability to complete MEG and MRI (e.g., claustrophobia or metal implants in the body); (3) Poor data quality; (4) Pregnancy or breastfeeding. The same exclusion criteria were used for the age- and gender-matched HCs, who had no history of headache and were free of physical and psychiatric disorders. Headache information (headache history, headache frequency, etc.) and clinical scales were collected before MEG acquisition. The clinical scale included Headache Impact Test -6 (HIT-6), Patient Health Questionnaire-9 (PHQ-9), Generalized Anxiety Disorder-7 (GAD-7), Pittsburgh Sleep Quality Index (PSQI), Visual Analogue Scale (VAS) and Montreal Cognitive Assessment (MoCA). The above scales assessed the intensity of headache impact, anxiety and depression symptoms, sleep quality, pain degree, and cognitive level of patients.

The study protocol was approved by the Institutional Review Committee of Beijing Tiantan Hospital of Capital Medical University (KY2022-044), which was registered on the https://www.clinicaltrials.gov (unique identifier: NCT05334927). All participants provided informed written consent according to the Declaration of Helsinki.

### MRI data acquisition

All participants were imaged with a 3.0 Tesla MR scanner (GE Healthcare, Milwaukee, WI, USA) at the Nuclear Medicine of Beijing Tiantan Hospital. Examinations were performed by a neuroradiologist who was unaware of the participant's diagnosis. Participants were asked to keep their heads and neck still, stay awake, and close their eyes, with tools to reduce noise and head movements. After checking the images, exclude images with quality problems. T1-weighted volumetric images were obtained by the 3D BRAVO sequence (coronal acquisition, the field of view (FOV) = 256 mm, acquisition matrix = 256, slice number = 192, flip angle = 15°, TR = 850 ms, TE = 320 ms, voxel size = 1 × 1 × 1.5 mm3).

### MEG data acquisition

The Elekta Neuromag 306-channel scanner (Elekta TRIUX ®) was used in this study to record neural activity at 2000 Hz with a low-pass filter set to 660 Hz. The Elekta Neuromag scanner with 306 channels (102 magnetometers and 204 gradiometers) was used in this study. The head position of the participants is detected by four (head position indicator) HPI coils, and re-recorded if the head movement was excessive during the scan. Then, the head position is digitally marked (Polhemus Fastrak®). About 300 points were marked on the nasion, anterior points in front of the ear points and scalp for MRI co-registration. Data acquisition was performed using a 2000 Hz sampling rate and a low-pass filter set to 660 Hz, while the participants’ electrooculogram and electrocardiogram were recorded. Resting-state MEG data were collected for five minutes for each participant. During the scan, participants were instructed to keep their heads and neck still, stay awake, and close their eyes.

### Preprocessing

After checking and excluding bad channels in the original data, the data were filtered using MaxFilter. The data sampling rate was reduced to 1000 Hz and 50 Hz line noise was removed, and finally a bandpass filter of 1-80 Hz was applied. In the independent component analysis (ICA), ocular and cardiac artifacts were marked and excluded. The cleaned data were then used to construct functional connectivity networks at the source level. Neural activity was filtered into five frequency bands: delta (1-4 Hz), theta (4-8 Hz), alpha (8-13 Hz), beta (13-30 Hz), gamma (30-80 Hz). At the source level, MRI images and MEG data were registered, and calculated the surface-based source space and inverse solution. Finally, dynamic statistical parametric mapping (dSPM) was used for source estimation [14].

**Fig. 1:**
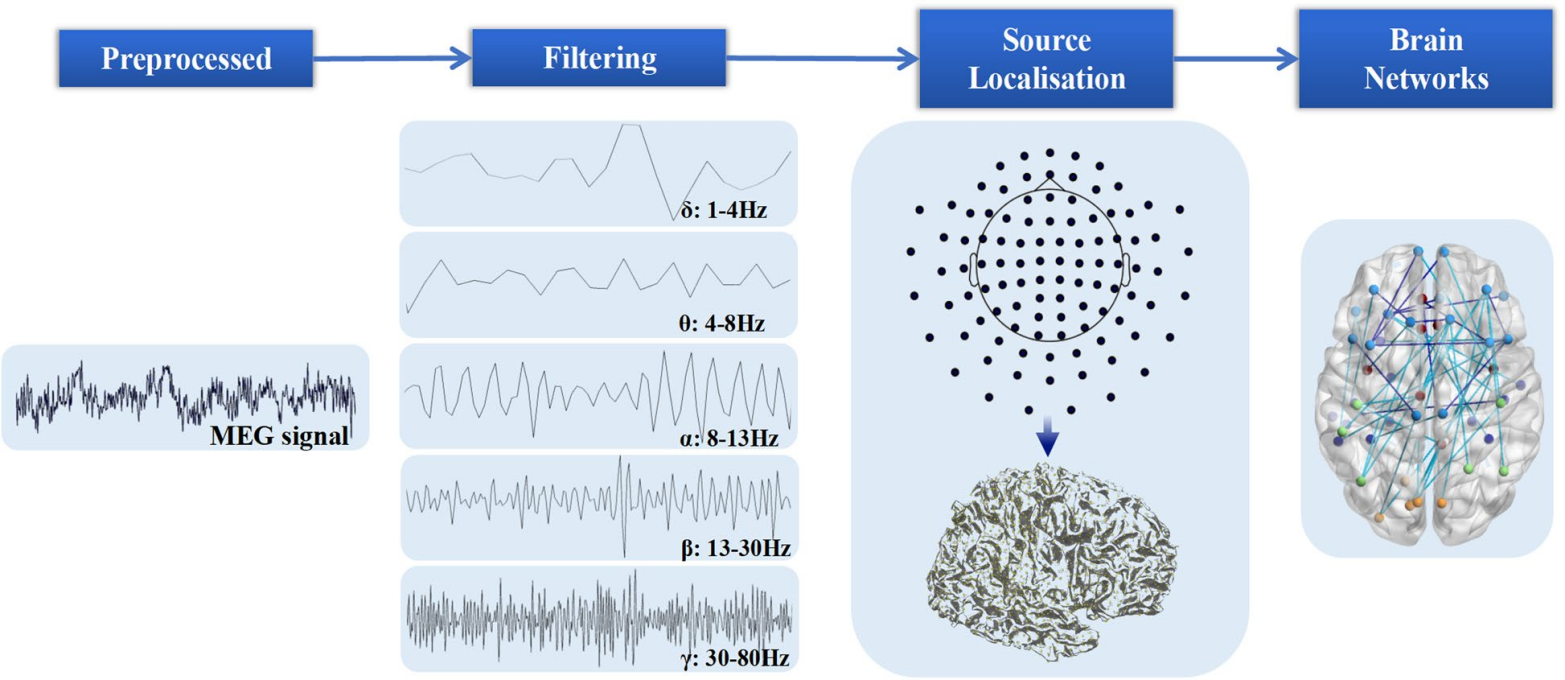
The pipeline of neural physiological signal to the construction of the brain network

Compute envelope correlations of orthogonalized activity as FC using pairwise and symmetric orthogonalization in source space. The procedure for symmetric orthogonalization in is: Extract inverse label data from raw; Symmetric orthogonalization; Band-pass filter; Hilbert transform and absolute value. According to the Desikan-Killiany Atlas, the brain was divided into 68 cortical regions (nodes). This power envelope time course was then correlated between brain region for each individual. These communication links between cortical regions (nodes) correspond to “edges” in a graph theory network model, and this specific approach for connectivity estimation shows greater repeatability than a wide range of other choices (Fig. 1) [15]. Network-based statistic (NBS) analysis was used to investigate which functional connections were significantly different between NDPH and HCs.

We computed the network topological parameters with graph theory that summarize the aspects of segregation and integration of a network. In this study, we focused on network nodal and global parameters including nodal clustering coefficient, nodal efficiency, nodal degree, global efficiency, local efficiency, shortest path length of a network. The complex network analyses were performed at a sparsity range from 0.05 to 0.39 with an interval of 0.01, and the area under curve (AUC) values under this range of sparsity were calculated for both global and nodal network parameters for statistical analyses. All network analyses were performed by Gretna (http://www.nitrc.org/projects/gretna/) and visualized by using BrainNet Viewer software (http://www.nitrc.org/projects/bnv/).

### Statistical analysis

The sample size was determined based on the available data and previous literature. Assuming no negative correlation between endpoints, a sample size of 75 cases (40 HC group and 35 NDPH group) would provide 90% power to reject the null hypothesis equal means at a two-sided alpha of 0.05. IBM SPSS 26.0 was used to perform the statistical analysis. Independent sample t-test and chi-square test were used to calculate the statistical difference of clinical data between groups. Continuous variables were tested for normality using the Kruskal–Wallis test; data conforming to a normal distribution were expressed as mean ± standard deviation, and otherwise as median with interquartile range. Categorical variables were expressed as numbers (percentages). Correlations between network parameters and demographic data were calculated using Pearson’s correlation, and false discovery rate (FDR) correction was performed. In NBS analysis and graph theory analysis, age and gender were used as covariables. Finally, the patients was grouped according to the headache location (unilateral and bilateral), the network topology parameters between the groups were compared. All statistical tests were two-tailed tests, with *P* < 0.05 indicating statistical significance.

## Results

### Clinical characteristics and demographics

**Fig. 2:**
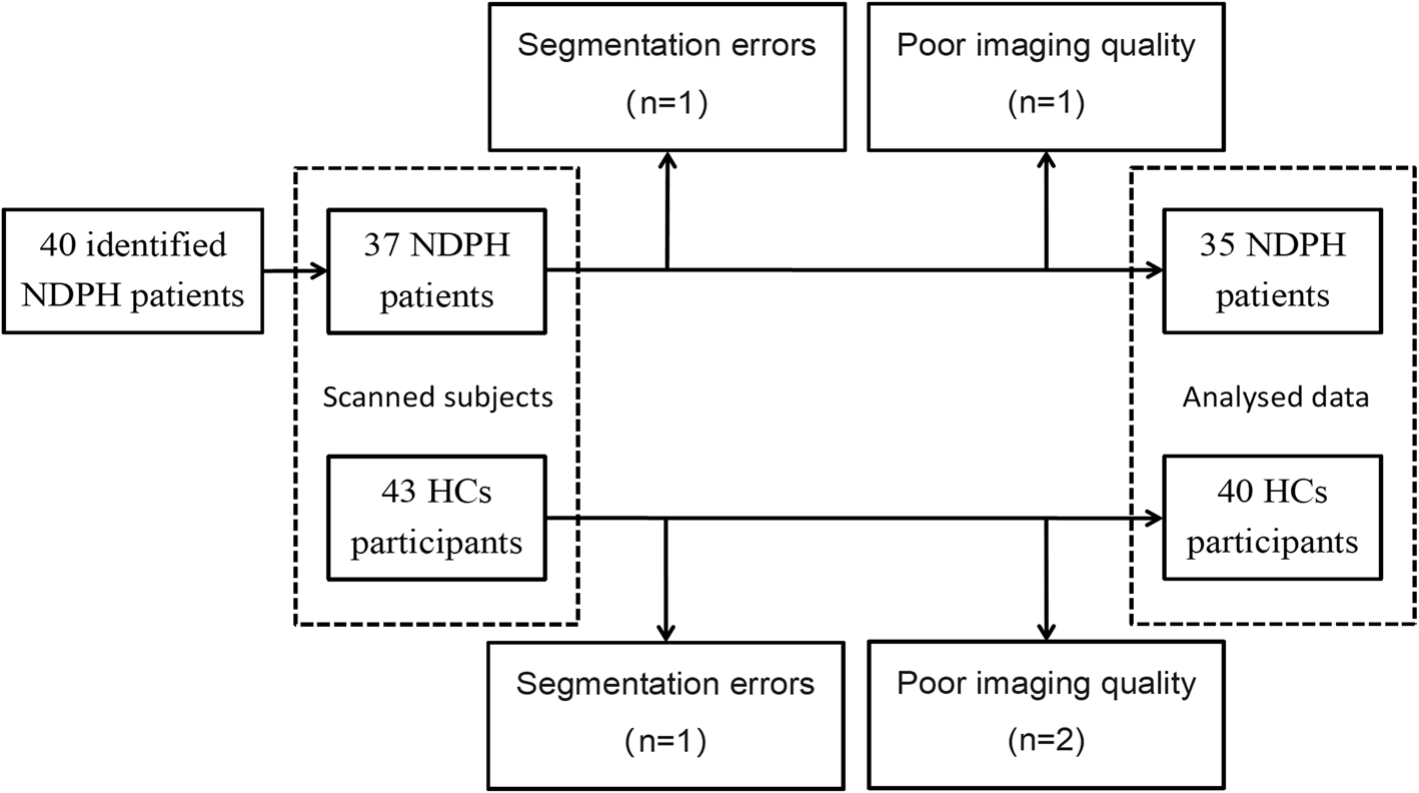
Illustration of the recruitment and exclusion process

This study included a total of 40 patients diagnosed with NDPH and 43 HCs, who were age- and gender-matched. Three patients dropped out of the study for personal reasons. Two patients and 3 HCs were excluded due to poor quality of data, segmentation errors (Fig. 2). Finally, thirty-five patients with NDPH (40.63 ± 15.65 years; 18 females) and 40 HCs (38.00 ± 13.00 years; 24 females) were included in this study. Of these patients with NDPH, 18 presented unilateral headache (51.5%). The clinical manifestations of the NDPH group were mainly nausea and vomiting (34.3%), photophobia (42.9%), and phonophobia (62.9%). The details are summarized in Table 1. All participants were right-handed and no SARS-CoV2 infection before developing headache.

**Table 1:** Participants’ demographics and clinical characteristics

	**Healthy Controls (*n* = 40)**	**NDPH (*n* = 35)**	***p*-value**
Age, years	38.00 ± 13.00	40.63 ± 15.65	0.430
Gender (male/female)	16/24 (40.0/60.0%)	17/18 (48.5/51.4%)	0.456
BMI (kg/m^2^)	22.41 ± 2.63	22.37 ± 2.96	0.947
Headache laterality, n (%)			
Unilateral	NA	18 (51.4%)	NA
Bilateral	NA	17 (48.6%)	NA
Location of headache, n (%)			
Frontal region	NA	15 (42.9%)	NA
Temporal region	NA	16 (45.7%)	NA
Parietal region	NA	14 (40.0%)	NA
Occipital region	NA	16 (45.7%)	NA
Periorbital region	NA	2 (5.7%)	NA
Nausea, vomiting, n (%)	NA	12 (34.3%)	NA
Photophobia, n (%)	NA	15 (42.9%)	NA
Phonophobia, n (%)	NA	22 (62.9%)	NA
Age at onset, years	NA	30.00 (19.00, 37.25)	NA
Disease duration, years	NA	2.00 (0.75, 7.00)	NA
VAS score (0–10)	NA	5.00 (4.00, 8.00)	NA
MoCA score (0–30)	NA	26.00 (21.00, 28.00)	NA
HIT-6 score (36–78)	NA	62.66 ± 8.70	NA
PHQ-9 score (0–27)	NA	9.00 (8.00, 16.00)	NA
GAD-7 score (0–21)	NA	7.77 ± 5.25	NA
PSQI score (0–21)	NA	9.31 ± 4.84	NA

Data are presented as mean ± standard deviation or as median [interquartile range, IQR]

*NDPH* new daily persistent headache, *NA* not applicable, *BMI* body mass index, *VAS* visual analogue scale, *MoCA* Montreal Cognitive Assessment, *HIT-6* Headache Impact Test-6, *PHQ-9* Patient Health Questionnaire-9, *GAD-7* Generalized Anxiety Disorder-7, *PSQI* Pittsburgh Sleep Quality Index

### Abnormal brain functional networks in patients with NDPH

**Fig. 3:**
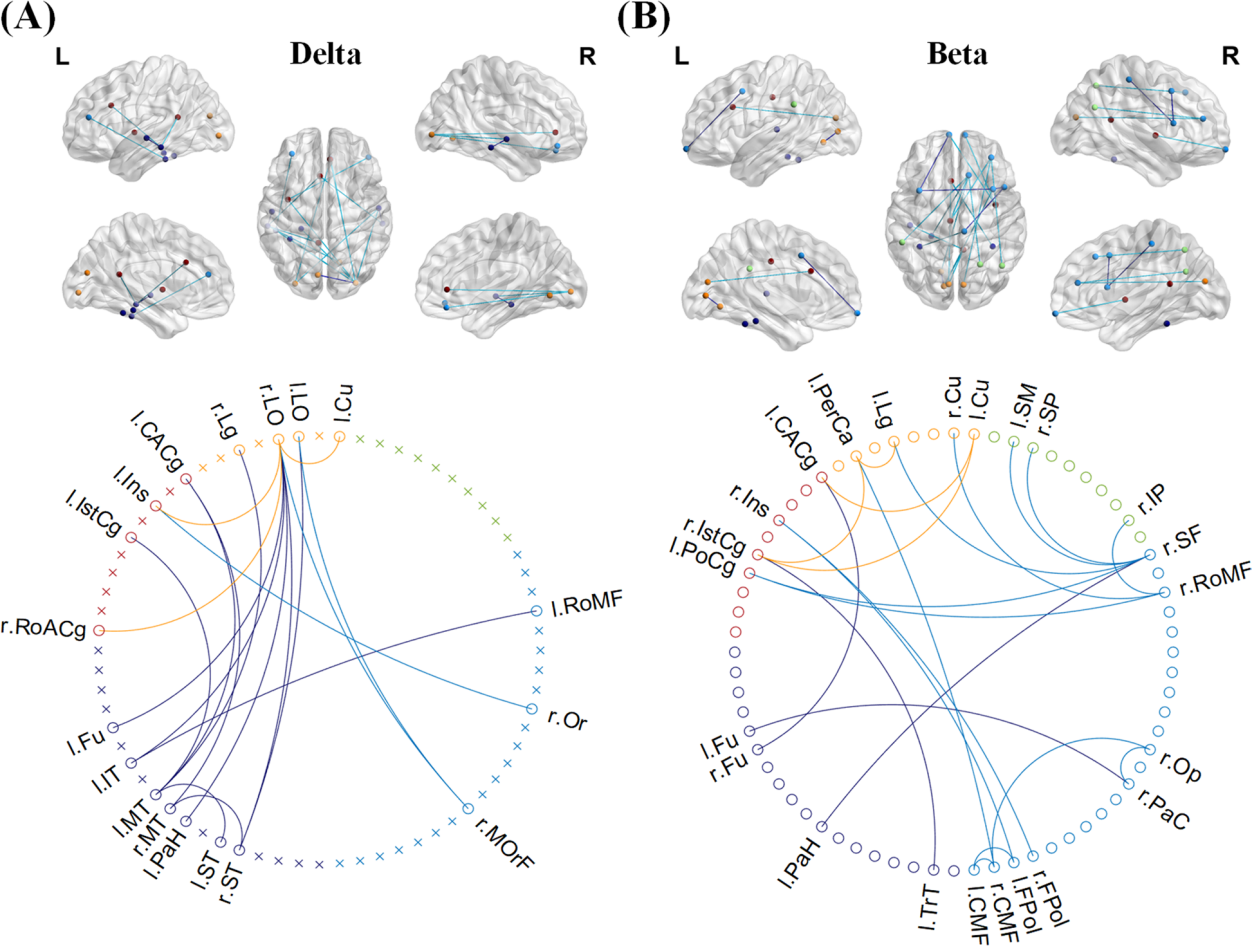
**A** Increased functional connections in patients with NDPH in the delta band. **B** Increased functional connections in NDPH patients in the delta band. In the diagram of the brain, light blue edges represent interlobar connections, and dark blue edges represent intra-lobar connections. Nodes of the frontal lobe (light blue), temporal lobe (dark blue), parietal lobe (green), orange (occipital), and cingulate cortex (brown) are marked in the connecting network. L, left; R, right; Cu, Cuneus; LO, Lateral occipital; Lg, Lingual; PerCa, Pericalcarine; CACg, Caudal anterior cingulate; Ins, Insula; IstCg, Isthmus cingulate; PoCg, Posterior cingulate; RoACg, Rostral anterior cingulate; Fu, Fusiform; IT, Inferior temporal; MT, Middle temporal; PaH, Parahippocampal; ST, Superior temporal; TrT, Transverse temporal; CMF, Caudal middle frontal; FPol, Frontal pole; MOrF, Medial orbitofrontal; PaC, Paracentral; Op, Pars opercularis; Or, Pars orbitalis; RoMF, Rostral middle frontal; SF, Superior frontal; IP, Inferior parietal; SP, Superior parietal; SM, Supra marginal

NBS analysis revealed increased FC in the lateral occipital cortex and superior frontal gyrus in the patient group compared to HCs. Analysis of the NBS revealed two single connected subnetwork, one with 18 nodes and 18 connections in the delta band and one with 23 nodes and 22 connections in the beta band, with the NDPH group having lower connection strength than HCs in both bands (*p* < 0.05). The abnormal connectivity was mainly distributed in the bilateral frontal, temporal, and occipital regions, including both long- and short-range connections (Fig. 3).

### Graph theory analysis

Group comparisons revealed that the global efficiency of the functional connectivity network in patients with NDPH were greater in the delta band compared to those of controls. Compared with HCs, the clustering coefficient of the left medial orbitofrontal cortex in patients with NDPH increased in the theta band. No significant network topological parameters difference was observed in other frequency bands. There was no difference in topological parameters between the two groups of patients with unilateral headache and bilateral headache.

### Correlation analysis

**Fig. 4:**
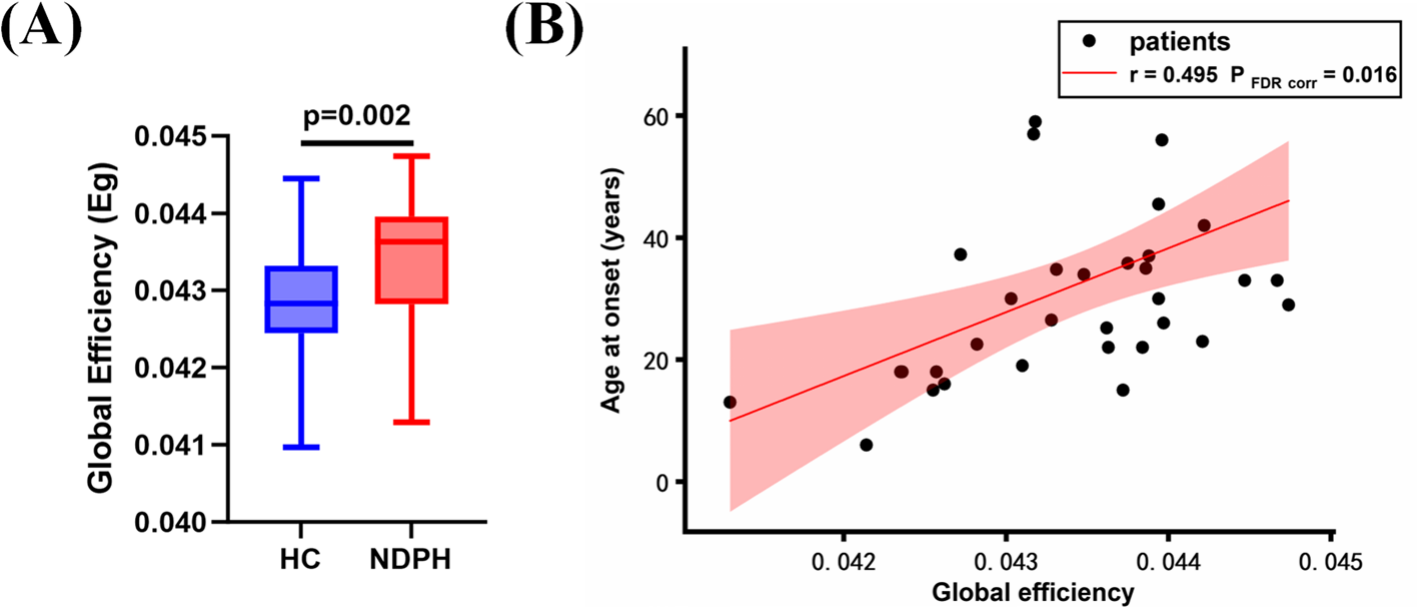
**A** Boxplot shows the group differences in the global efficiency of networks in the delta band. The bars and error bars represent the fitted values and the standard deviations, respectively; **B** Correlation between global efficiency and age at onset. HC, healthy control; NDPH, new daily persistent headache

**Fig. 5:**
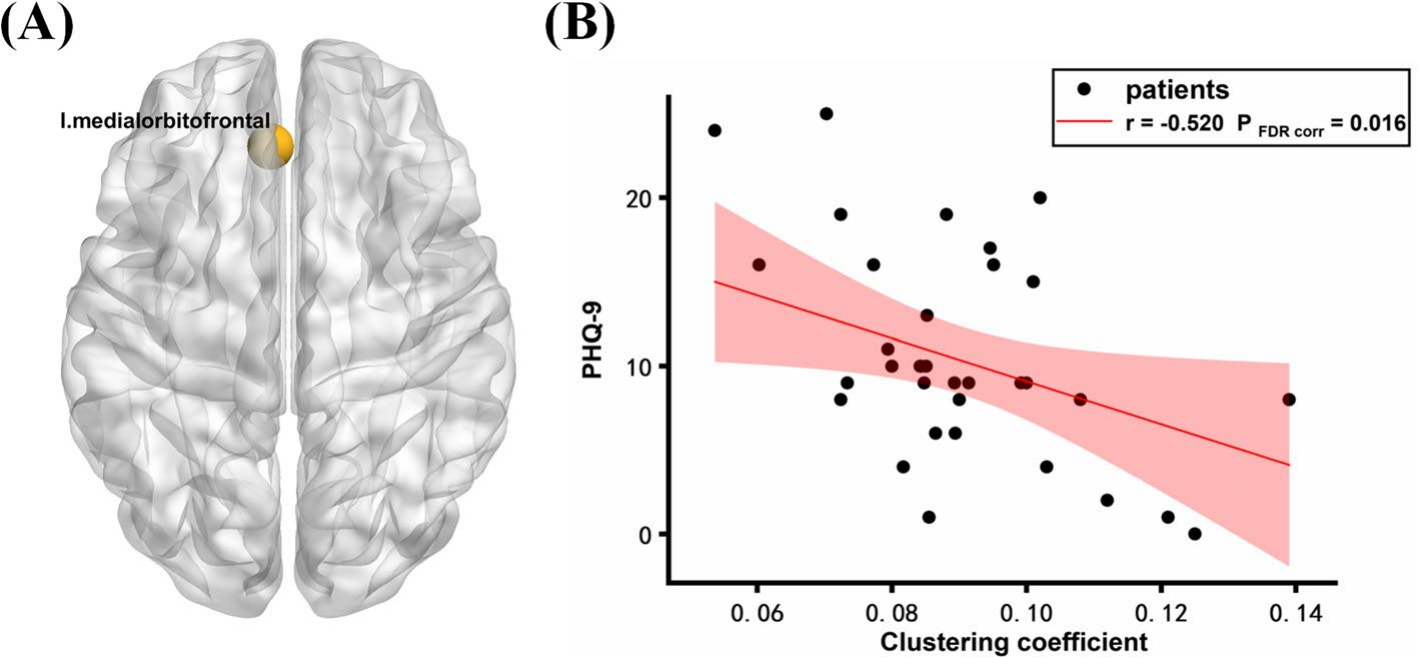
**A** In the theta band, the medial orbitofrontal cortex with significantly lower nodal clustering coefficient in the NDPH group compared to HC group; **B** Correlation between clustering coefficient of medial orbitofrontal cortex and PHQ-9 scores. HC, healthy control; NDPH, new daily persistent headache

Clinical measures (age at onset, disease duration, VAS, MoCA, HIT-6, PHQ-9, GAD-7, PSQI, and MIDAS) did not correlate significantly with the connectivity strengths as described by a global mean of the FC. We found that the age at onset had a positive correlation with global efficiency in the delta band (*p* = 0.016, *r* = 0.495) (Fig. 4). A significant negative correlation was found between the PHQ-9 and the clustering coefficient of the left medial orbitofrontal cortex in the theta band (*p* = 0.016, *r* = -0.520) (Fig. 5). There was no statistically significant correlation between the 2 parameters (global efficiency; clustering coefficient of the left medial orbitofrontal cortex) in the graph theory of NDPH patients and other clinical measures.

## Discussion

In this study, we innovatively used MEG to directly observe the neural magnetic signals of the participants, and observed the correlation between various cortical brain regions through the method of oscillating envelope correlation and graph theory, and analyzed the brain topological properties of the participants. Compared with HCs, patients with NDPH showed a broad increase in FC in the delta and beta bands, especially in the frontal, temporal, and occipital lobes. The distribution of FC and graph-theory analysis indicated the differences in neural networks between the NDPH and HC group in some frequency bands. Those abnormalities may have considerable mechanistic importance given the key role of neural oscillations in the long-range synchronization of information across the brain and might contribute to the development of accessible multimodal biomarkers.

### Abnormal brain functional networks of NDPH

Previous studies have found that MEG resting-state activity exposes significant cortical activity changes among patients with NDPH [16]. In the delta band, our results suggest that NDPH exhibit different connective patterns between the lateral occipital cortex and the temporal lobe compared to HCs. The occipital lobe is the visual processing center containing most of the anatomical region of the visual cortex. In our previous studies, we found that patients with NDPH had altered brain perfusion and abnormal activation of the visual system [17, 18]. Changes in the visual cortex have also been observed in other headache disorders. Studies of interictal migraine have reported that abnormal FC in the occipital lobe is involved in the pathophysiological process of headache [19]. In a magnetic resonance spectroscopy (MRS) study, cortical excitability was altered in patients with migraine, and abnormal levels of multiple neurotransmitters were observed in the occipital cortex [20–22]. Meanwhile, migraine-like animal models suggest that the trigeminal nucleus caudalis projects indirectly to the primary visual cortex involved in the emotional/motivational aspects of pain perception and that the primary and secondary visual cortex are involved in photophobic circuits for migraine-related pain [23]. Based on the above evidence, abnormal network patterns and neurotransmitter changes in the occipital lobe may be associated with headache. In the NDPH group, 42.9% of patients were associated with photophobia. We hypothesized that the abnormal network pattern of the lateral occipital lobe may be involved in the occurrence of photophobia.

In addition, extensive increased FC of the temporal and occipital lobe was observed in this study. The temporal lobe is involved in processing sensory input into derived meanings for the appropriate retention of visual memory, language comprehension, and emotion association [24, 25]. Imaging studies of headache showed abnormal cerebral blood flow in the bilateral frontal and temporal lobes, and the cerebral blood flow in the temporal lobe was correlated with the severity of headache [26]. Similarly, in MEG studies of migraine patients, it was observed that patients had significantly enhanced connections from the frontal to temporal cortex in response to negative emotional stimuli [27]. In our study, more than half of the patients were accompanied by phonophobia (62.9%). Some studies have shown that enhanced regional functional activity of the superior temporal gyrus is presumed to be related to auditory information processing in patients with vestibular migraine. We hypothesized that the increase of FC in temporal lobe may be involved in the pathophysiology of phonophobia in NDPH [28]. In summary, our results suggest that the increase of FC in the temporal lobe may be involved in auditory information processing and emotional memory regulation in patients with NDPH.

In the beta band, the NDPH had significantly increased FC in the frontal lobe. A recently published paper has revealed that the functional connections of frontal lobe, amygdala, and insula were changed in patients with NDPH compared with HCs [29]. MEG analysis showed that patients with NDPH had structural changes and abnormal high-frequency cortical activity in both the frontal and temporal lobes [16]. The frontal lobe involves pain regulation, cognitive control, and executive function, and thus is a primary candidate for dysfunction in many neurodevelopmental and neuropsychiatric disorders [30, 31]. Increased FC in frontal lobe may support the theory of central hyperexcitability in NDPH. These findings may facilitate to develop new therapeutic strategies for migraine. For example, in the following studies, repetitive transcranial electrical stimulation (rTMS) can be applied to patients with NDPH to inhibit frontal neuron excitation through low-frequency rTMS, and clinical efficacy can be observed to determine the role of frontal lobe in the pathophysiology of NDPH [32, 33].

### Topological characteristics of NDPH

In this study, we used graph theory for the first time to analyze the functional network topological properties of NDPH patients. Compared to HCs, the global efficiency of the functional connectivity network in patients with NDPH was increased in the delta band. The global efficiency mainly characterize the global information transmission capacity of brain network, and higher global efficiency of a network reflect faster information transfer between network nodes [34]. Different levels of visual perceptual skills are associated with specific modifications in global efficiency, and higher global efficiency may imply increased hemispherical asymmetry [35]. Thus, these findings about abnormal global efficiency may support the speculation that the brain networks in patients with NDPH tend to be randomized with increased global integration to obscure the role of key nodes and reduce the modularity of the network.

Our study showed that the clustering coefficient of the left medial orbitofrontal cortex in patients with NDPH increased in the theta band. The clustering coefficient of a node represents the ratio of all existing connections between the “neighbors” of a node (nodes that are one-step away) and the maximum possible number of edges between the neighbors. The orbitofrontal cortex was defined as the part of the prefrontal cortex that receives projections from the medial dorsal nucleus of the thalamus [36–38]. Functional and structural prefrontal cortex abnormalities have been reported in major depressive disorder. Patients with depression have been linked to decreased activation in ventral and dorsal striatum as well as middle frontal gyrus, but hyperactivation in the prefrontal cortex [39, 40]. The mid-anterior orbitofrontal cortex has been found to consistently track subjective pleasure in neuroimaging studies [41]. We observed that patients with NDPH generally had mild to moderate depression, which may be related to decreased local integration of the medial orbitofrontal cortex.

### Clinical correlation analysis of NDPH

To identify the clinical significance of aberrant MEG parameters in NDPH, we further calculated the correlations between the aberrant MEG parameters and clinical characteristics in NDPH. We found that the age at onset had a positive correlation with global efficiency in the delta band. And a significant negative correlation was found between the PHQ-9 and the clustering coefficient of the left medial orbitofrontal cortex in the theta band. By studying the developmental changes in functional brain networks from birth to adolescence, Gozdas found that the global efficiency increases with age [42]. This may explain why the greater the age of onset in patients with NDPH, the greater the global efficiency. Functional and structural changes in the medial orbitofrontal cortex, a brain region closely related to emotion, have been reported in depressed patients [43]. Our results suggest that the medial orbitofrontal cortex may play an important role in the development of depression in patients with NDPH. It is worth noting that our previous study also found that the cortical thickness of the left rostral middle frontal gyrus was reduced, so it is necessary to verify the important role of the frontal lobe, especially the prefrontal cortex, in patients with NDPH [16]. There were no correlations between the aberrant MEG parameters and other clinical characteristics in NDPH.

### Study limitations and future directions

We noted that this study has some limitations. The first limitation of this study is the small number of participants, and our results still need to be validated in more data. Second, this study lacked longitudinal data of participants to observe neuromagnetic source activity during NDPH. Finally, our study found a close relationship between the degree of depression in NDPH and the medial orbitofrontal cortex. Therefore, it is necessary to distinguish various comorbidity subgroups of NDPH further and conduct clinical intervention to seek new ways to treat headache in the future.

## Conclusions

This study suggests that the FC and topology of NDPH in brain networks may be altered and the cortex is overexcited. The increased FC in lateral occipital cortex and superior frontal gyrus under resting-state MEG may be one of the imaging features of NDPH. In addition, the frontal lobe (especially the medial orbitofrontal cortex) may play an important role in NDPH and is closely related to the degree of depression in patients with NDPH. These findings may contribute to developing new treatment strategies for NDPH, which can be further clinically validated by non-invasive neuroregulatory techniques (such as rTMS) in the future.

## Data Availability

The data that support the findings of this study are available from the corresponding author, upon reasonable request.

## Abbreviations

AUC: Area under curve
dSPM: Dynamic statistical parametric mapping
NDPH: New daily persistent headache
EEG: Electroencephalography
fMRI: Functional magnetic resonance imaging
FC: Functional connectivity
FOV: the field of view
FDR: False discovery rate
GAD-7: Generalized Anxiety Disorder-7
HCs: Healthy controls
HIT-6: Headache Impact Test-6
HPI: Head position indicator
ICA: Independent component analysis
MEG: Magnetoencephalography
MRI: Magnetic resonance imaging
MoCA: Montreal Cognitive Assessment
MRS: Magnetic resonance spectroscopy
NBS: Network-based statistic
PHQ-9: Patient Health Questionnaire-9
PSQI: Pittsburgh Sleep Quality Index
rTMS: Repetitive transcranial electrical stimulation
VAS: Visual Analogue Scale
